# The dual impact of tobacco spending: crowding out essentials and crowding in addictive behaviors

**DOI:** 10.1038/s41598-025-08648-1

**Published:** 2025-07-09

**Authors:** Elvina Merkaj, Drini Imami, Jeffrey Drope

**Affiliations:** 1 Polytechnic University of Marche, Ancona, Italy; 2 Agriculture University of Tirana, Tirana, Albania; 3 Economics for Health, Johns Hopkins Bloomberg School of Public Health, Baltimore, USA

**Keywords:** Tobacco spending, Crowding out effect, Albania, Health care economics, Health policy, Quality of life

## Abstract

Tobacco use, a major preventable risk factor for premature death and morbidity, profoundly shapes individual and household decision-making. Tobacco spending in lower-income economies exacerbates economic strain of the households. To accommodate tobacco costs, many households are forced to reduce essential expenditures on health and education—a phenomenon known as the crowding out effect. This study uses data from Albania’s national Household Budget Survey and applies a three-stage least squares method with instrumental variables to estimate Engel curves to shed light on how tobacco use influences household financial decisions. The findings reveal that tobacco spending significantly reduces expenditures on essential needs such as health, education, housing, and clothing. Additionally, tobacco use shows a positive correlation with alcohol spending, revealing another negative influence of an addictive behavior on household resource allocation. The results emphasize the urgent need for stronger tobacco control measures promote better economic decision-making within households. Such measures would enhance public health, improve economic stability, and encourage more equitable resource distribution.

**Supplementary Information:**

The online version contains supplementary material available at 10.1038/s41598-025-08648-1.

## Introduction

Major causes of illness and premature death are often linked to various personal addictions and daily behavioral choices. Previous studies have shown that eight of the nine leading causes of death, are related to lifestyle decisions^1,2^. Tobacco use is widely demonstrated to be among the largest preventable risk factors for premature death and increased morbidity, and in some countries, the most impactful. It is a major risk factor for non-communicable diseases such as cancers, lung and cardiovascular diseases, and diabetes^3^.

Tobacco consumption is a deeply rooted addiction that poses significant health risks while profoundly and often adversely influencing individual and household economic decision-making. Behavioral economics offers insights into tobacco consumption behaviour. The addictive nature of tobacco causes individuals to overvalue immediate gratification from smoking while undervaluing its long-term health and financial consequences^4^. This dynamic is often set in motion during youth, especially considering that most smokers begin using tobacco in childhood or adolescence. It is well documented that youth typically have a stronger present bias and weaker will power than older individuals so the lure of trying something forbidden and seemingly mature, particularly to impress and/or fit in with peers increases their likelihood of experimentation^5^. But again, the real problem lies in the highly addictive nature of nicotine because the individual underestimates the overall harm and how difficult it will be to quit in the future^6^.

Addictions like smoking influence how individuals and households allocate limited resources, frequently leading to suboptimal economic choices^7^. Households often redirect scarce financial resources toward sustaining this addiction, resulting in reduced spending on critical needs such as education, healthcare, housing etc^8^. This behavior is known as the "crowding-out effect" in economic literature, wherein consumption of one good or service (in this case, tobacco) displaces spending on other important goods and services^9,10^. The effect is particularly harmful for low^11^ and even some middle-income households, where the financial strain of maintaining a smoking addiction puts considerable pressure on already tight budgets. Smoking is not evenly distributed across society, but rather shaped by broader socioeconomic factors that influence both the prevalence and intensity of tobacco use^12^. Furthermore, the adverse health effects of smoking contribute to lower household income due to increased morbidity and mortality rates, which reduce economic productivity and further exacerbate the financial strain on these households^13^. In this context, the crowding-out effect creates a vicious cycle, where the economic burdens of smoking lead to further deprivation in critical areas, limiting opportunities for social mobility and long-term well-being, especially for vulnerable households.

One major consequence of tobacco consumption is its tendency to crowd out educational opportunities^9,10^, meaning that financial resources that could be used for education are instead spent on sustaining the smoking addiction. This shift in priorities results in fewer resources available for essential educational needs, such as school supplies, tuition, or extracurricular activities. This may lead to limited social mobility for household members, particularly children of smokers. As a result, these children often may have fewer opportunities for advancement due to the negative impacts of parental smoking on their educational outcomes. In addition, smoking within the household perpetuates a harmful cycle, where detrimental addictions are passed down to future generations. Research, including studies conducted in Albania, consistently demonstrates that parents who smoke increase the likelihood of their children smoking^14^. This transmission occurs not only through direct exposure and behavioral modelling but also through indirect pathways, as smoking can hinder a child’s educational progress and future potential.

Tobacco consumption is strongly and positively correlated to alcohol consumption^15^. Research indicates a positive association between alcohol and cigarette consumption, as they are often shown to be complementary products^16–18^. Not surprisingly, individuals who quit smoking often also reduce their daily alcohol consumption^19^. Similarly, tobacco use is associated with greater spending on alcohol-related services, such as bars, restaurants, and entertainment venues^9,10,20^. This connection extends to diet as well, as smokers and drinkers are more likely to make poor food choices (e.g., more processed foods), leading to declines in healthy eating habits and nutrient intake^21^.

In Albania, the interplay between tobacco consumption as an addictive behavior and its consequences is highly pronounced. Around 25% of adults smoke, and especially men (43%)^22^. Higher male smoking rates start in childhood – previous studies have shown that male pupils have higher odds to smoke than females^14,23^. Smoking is a leading risk factor for disease and premature death in Albania, responsible for 25% of male deaths and 9.1% of female deaths annually, totalling more than 4,000 deaths per year. It is one of three main contributors to Albania’s disease burden^24^, which implies a significant economic cost. This is particularly problematic as Albania is among the poorest countries in Europe. In 2022, 20.6% of Albanians were at risk of poverty, while 33.2% faced severe material deprivation^25^. Smoking behavior is deeply rooted in social norms and cultural practices, yet its consequences extend far beyond individual health. Previous research in Albania shows that nearly 13,000 households, including 60,000 people (10,000 children), are pushed below the poverty line due to tobacco-related costs, worsening their economic situation^26^. For these families, the addictive nature of tobacco consumption is not a matter of choice but a reflection of behavioral inertia, wherein the immediate satisfaction derived from smoking takes precedence over more prudent financial decisions.

The profound influence of tobacco consumption on household economic decision-making is not unique to Albania. Studies from other countries and regions, including India^9^, Turkey^27^, Vietnam^28^, Serbia^29^, and Montenegro^30^, consistently reveal how tobacco use constrains household budgets. While findings may vary somewhat by socioeconomic and cultural context, overall, these studies provide strong evidence that smoking as a highly addictive behavior undermines households’ ability to allocate resources effectively by reducing spending on essential goods and services.

Albania’s unique socioeconomic and cultural landscape, however, remains underexplored, leaving critical gaps in understanding how this dynamic plays out within the country. This study aims to address these gaps by investigating how the addictive nature of tobacco consumption influences household spending decisions in Albania. Using data from Albania’s Household Budget Survey (HBS), this study analyses the crowding out effects of tobacco spending in the country. The results show that tobacco spending decreases expenditures on healthcare, education, housing, and clothing while increasing alcohol spending. By examining spending patterns across income groups, the study sheds light on how smoking behaviors affect different segments of society.

The findings underscore the importance of addressing tobacco use not just as a public health issue but also a behavioral and economic challenge. Tobacco use not only harms health but also diverts spending from essential goods, disproportionately affecting children by limiting their access to education and other necessities. This reduces human capital accumulation and perpetuates economic struggles for future generations. Comprehensive tobacco control measures—including higher excise taxes, targeted public awareness campaigns, and support for smoking cessation—are crucial to breaking the cycle of tobacco consumption. By reducing the financial strain imposed by this behavior, such policies can help households make more optimal economic decisions, fostering both individual well-being and broader economic resilience.

## Theoretical framework and econometric model

### Household utility maximization

This study is grounded in the theory of household utility maximization, which posits that households make spending decisions to achieve the highest possible utility within the constraints of their budget. Household preferences can be represented by a utility function, reflecting how different goods contribute to overall utility, subject to a budget constraint. The demand for each good is thus a function of prices, income, and household-specific characteristics.

Let U = U(x_1_,…, x_n_: h) represent the household utility function, where ${x}_{i}$ denotes the quantity consumed of the i-th good, and ***h*** is a vector of household characteristics. The household maximizes utility subject to a budget constraint, where total expenditure is Y, and the prices of all goods are given by p_1_,…, p_n_. The utility maximization problem can be written as:

$$Max U = U\left( {x_{1} , \ldots , x_{n} ;{\varvec{h}}} \right) \;\;s.t. \mathop \sum \limits_{i = 1}^{n} p_{i} x_{i} = Y$$

The solution to this problem yields the unconditional marshallian demand functions for each good – or the optimal quantity of each good that a household will consume – dependent on total income Y, prices P = (p_1_,…,p_n_) and the household characteristics ***h***:

$$x_{i} = f^{i} \left( {p_{1} , \ldots , p_{n} , Y;\user2{ h}} \right) = f^{i} \left( {P, Y;\user2{ h}} \right),\quad \forall i = 1, 2, \ldots , n$$

### Conditional demand framework

Tobacco consumption’s addictive nature introduces a dimension that alters standard economic decision-making dynamics. As an addictive good, tobacco consumption often takes precedence in household budgets, reducing the resources available for other goods and services. Therefore, to specifically analyze the impact of tobacco consumption, this study employs the conditional demand framework^31^, as in^9^. Here, tobacco expenditure is treated as a fixed allocation, transforming the household’s decision-making process for other goods. In the presence of addictive consumption, such as tobacco, a portion of the budget is pre-allocated, reducing the effective income available for other goods. This constraint alters the utility maximization process, by reducing the available income for all other goods and creating a framework for understanding the crowding-out effects of tobacco.

Suppose tobacco is the n-th good. The household pre-allocates a certain amount of its budget, p_t_* x_n_ to tobacco, where *p*_*t*_ is the price of tobacco and x_n_ is the fixed quantity consumed. Thus, the remaining income available for the consumption of the other n − 1 goods is given by *M* = *Y − p*_t_* x_n_. The new utility maximization problem becomes:

$$Max U = U\left( {x_{1} , \ldots , x_{n} ;{\varvec{h}}} \right)$$

$$s.t. \mathop \sum \limits_{i = 1}^{n - 1} p_{i}  x_{i} = M$$

$$s.t. x_{n} = \overline{{x_{n} }}$$

where ${\text{x}}_{{\text{n}}} = {\overline{\text{x}}}_{{\text{n}}}$, represent the household’s predetermined tobacco allocation and ***h*** is a vector of household characteristics. Solving this maximization problem for the remaining n − 1 goods yields the conditional demand functions, which can be written as:

$$x_{i} = g^{i,n} \left( {p_{1} , \ldots , p_{n - 1} , x_{n} , M;\user2{ h}} \right),\quad \forall i = 1, 2, \ldots , n - 1$$

Here, the function g^i,n^ represents the conditional demand function for the *i-th* good, conditional on the consumption of the *n-th* good (in this case, tobacco). This framework allows for an examination of how spending on essential goods changes from addictive tobacco use.

The use of conditional demand functions offers several advantages^31,32^. By holding the consumption of tobacco fixed, we isolate the impact of tobacco spending on the demand for other goods. This framework is particularly useful when studying households with varying levels of tobacco consumption, as it allows us to test whether the consumption patterns of tobacco users differ significantly from non-users.

Scholars^33^, further expands on this by introducing the concept of consumer separability, which tests whether the preferences of tobacco users and non-users differ fundamentally. In this context, scholars augment the conditional demand function with a binary indicator *d*, where d = 1 if the household spends on tobacco and d = 0 if they do not. The goal is to assess whether this binary variable significantly influences the demand for other goods:

$$x_{i} = g_{i} \left( {p_{1} , \ldots , p_{n - 1} , x_{n} , d, M; {\varvec{h}}} \right),\quad \forall i = 1, 2, \ldots , n - 1$$

where p_1_,…, p_n-1_ are the prices of good 1 through n-1, M is non-tobacco expenditures, h is household characteristics and d is a dummy if a household spends on tobacco.

If *d* is statistically significant, this would suggests that tobacco use influences not only the available budget (income effect) but also alters the relative demand for other goods (substitution effect), thereby rejecting the weak separability assumption. Under weak separability, tobacco consumption should affect the total budget but leave the structure of demand for other goods unchanged.

For example, if two households with similar income differ only in that one spends on tobacco and the other does not, and both allocate their remaining income similarly across food, education, and health, this supports weak separability. Conversely, significant differences in their allocation patterns would imply that tobacco use modifies underlying preferences.

## Methods

### Data

To estimate the crowding out effects of tobacco consumption, this study uses 2017 Household Budget Survey (HBS) data. The HBS is a nationally representative survey, conducted by the Statistical Office of Albania (INSTAT). The survey is used for monitoring national expenditure trends. It is a relatively standardized instrument – the HBS is conducted in all European countries with a comparable methodology and coordinated by Eurostat. Importantly, the HBS is the only survey in Albania that provides detailed information on household expenditures, other economic and socio-demographic characteristics, and population weights needed for the estimation of the effects of tobacco expenditures on other consumption. The total number of households that participated in HBS during 2017 was 7,518. About 38 percent of these households have tobacco expenditures. In line with the Classification of Individual Consumption According to Purpose (COICOP) developed by the United Nations Statistics Division, household expenditures in HBS are divided into 12 mutually exclusive and exhaustive commodity groups. HBS data allow for further differentiation within the 12 groups, and this feature is utilized in this research to differentiate between tobacco and alcohol expenditures within the COICOP group 2 – Alcoholic beverages and tobacco. Therefore, a total of 13 mutually exclusive and exhaustive expenditure variables are used for the estimation of the crowding out effect in this study. The main goal is to estimate the effect of tobacco spending on the expenditure for the other 12 commodity groups.

### Econometric model

Empirically, the analysis uses the Quadratic Almost Ideal Demand System (QUAIDS)^34^. The QUAIDS model is preferred over the standard Almost Ideal Demand System (AIDS) model in this context because, by incorporating quadratic income terms, it allows for non-linear Engel curves, capturing more realistic household consumption behavior across income levels. While the AIDS model assumes a linear relationship between the log of income and expenditure shares—implying constant marginal budget shares and income elasticities—this assumption often fails in real-world settings, particularly in low- and middle-income countries. In contrast, QUAIDS introduces a quadratic term in log income $\left( {\ln M_{j} } \right)^{2}$, which allows the model to reflect non-constant income effects and nonlinear demand responses^34^. This is crucial for distinguishing between goods that behave as luxuries at lower income levels and necessities at higher levels. By accounting for these nonlinearities, QUAIDS offers the flexibility required to model heterogeneous consumption patterns, making it particularly well suited for analyzing crowding-out effects of tobacco spending across different income groups^9,35^.

Thus, incorporating household characteristics (h) and conditioning expenditures on tobacco (p_t_*x_n_), we estimate the following conditional Engel curves for 12 broad categories of goods and services:

1 $$w_{ij} = \beta_{0i} + \beta_{1i} tob\_\exp_{j} + \beta_{2i} \ln M_{j} + \beta_{3i} \left( {\ln M_{j} } \right)^{2} + \gamma_{i} {h_{j}}{^\prime} + u_{ij}$$

In this equation, $w_{ij }$ represents the budget share of the i-th product group in the j-th household’s total expenditures, net of tobacco spending. The term *tob_exp* denotes the household’s expenditure on tobacco, while M represents the total household expenditure after deducting the expenditures for tobacco spending. The vector *h* contains household characteristics such as household size, the number of children under 14 years, the number of elderly members (aged 65 or older), the maximum education years within the household, number of employed or self-employed members within the household, region dummies, and residence type (rural or urban). Finally, u_ij_ is the error term in the demand equation, capturing unobserved factors that affect the budget share of each product group per household. The inclusion of quadratic income terms $\left( {\ln M_{j} } \right)^{2}$ in the equation allows for variations in preferences across different income levels, as suggested by^9^. This feature enables the model to distinguish between goods that may be considered luxuries at lower income levels and necessities as income rises, offering a more nuanced understanding of household consumption behavior. For instance, food may dominate the household budget at low incomes, while the budget share for discretionary goods such as education or entertainment rises with income^36^.

The key coefficient of interest in the equation is $\beta_{1i}$, which estimates the crowding out effect of tobacco expenditures. If $\beta_{1i}$ is negative and statistically significant, it indicates that an increase in tobacco spending reduces the budget share allocated to the i-th product group, confirming a crowding-out effect. In this case, higher tobacco consumption would lead to lower expenditure on other goods, reflecting a trade-off between spending on tobacco and other household needs. Conversely, if $\beta_{1i}$ is positive, this suggests that tobacco consumption and spending on the i-th good are complementary. In this scenario, households that spend more on tobacco may also allocate a larger budget share to other related goods.

To further explore the interaction between tobacco consumption and household spending on other goods, we test for consumer separability. This involves augmenting the conditional demand function with a binary variable d, which indicates whether the household has positive tobacco expenditure (i.e., d = 1 for households that spend on tobacco, and d = 0 for those that do not). This test allows us to examine whether the preferences of tobacco-using households differ fundamentally from those of nontobacco ones.

To formally account for these potential preference differences, we extend Eq. (1) to incorporate the binary variable d, which allows for the separate estimation of preferences for tobacco users and non-users. The extended model can be expressed as follows (Please refer to Eq. 1 for variable definitions.):

2 $$w_{ij} = \beta_{0i} + \beta_{0di} d_{j} + \beta_{1i} tob\_\exp_{j} + (\beta_{2i} + \beta_{2di} d)\ln M_{j} + (\beta_{3i} + \beta_{3di} d)\left( {\ln M_{j} } \right)^{2} + \gamma_{j} h_{j}{^\prime} + u_{i}$$

This model allows to estimate the impact of tobacco consumption on the budget shares of other goods by accounting for potential heterogeneity between tobacco users and non-users.

### Estimation of the model: key challenges and solutions

When estimating the model to analyze the crowding-out effect of tobacco consumption, several important methodological challenges must be addressed.

The first challenge arises from the potential endogeneity of key variables such as tobacco expenditure (tob_exp) and non-tobacco expenditures (M) in the equation, primarily due to simultaneity. Households allocate their total budget between tobacco and other goods simultaneously. An increase in tobacco spending directly reduces the resources available for other goods, and vice versa. This simultaneity implies that both tobacco expenditures and M are jointly determined with the dependent variable and influenced by the same household-level preferences and constraints. Consequently, these variables are likely to be correlated with the error term in the model 1 (To empirically assess this endogeneity, we applied the GMM C-statistic test, which confirmed the endogeneity of the variables).

Such correlation results in endogeneity, which arises when explanatory variables are not independent of the error term. This violates a fundamental assumption of Ordinary Least Squares (OLS)—that regressors must be exogenous, or uncorrelated with the disturbance term^37^. When this assumption is breached, OLS estimates become biased and inconsistent, undermining their validity for causal inference. A standard solution to this problem is the use of instrumental variables (IV) estimation, which requires identifying exogenous variables that are correlated with the endogenous regressors but uncorrelated with the error terms^38,39^.

Research on the crowding-out effect of tobacco spending gained momentum around 2008, beginning with a study based on household expenditure data from India^9,37^ and was subsequently expanded by numerous scholars in the years that followed (among others Turkey^27^, Vietnam^28^, Serbia^29^, and Montenegro^30^). Additionally, investigations in Indonesia, Colombia, South Africa and other low- and middle-income countries (LMICs)^40–44^ explored the crowding-out phenomenon using alternative methods (see^35^ for more details). In this study, following above literature, we use total expenditures as an instrument for total expenditures excluding tobacco (M). Additionally, we use household gender ratio (the ratio of adult women to men) and adult ratio as instruments for tobacco expenditures. This choice is grounded in the well-documented observation that smoking prevalence is typically much higher among men than women^9,27^, a pattern also seen in Albania. The assumption here is that the gender ratio is correlated with tobacco expenditure but uncorrelated with budget shares on other goods. To strengthen the instrument set for tobacco expenditures, we also use average aggregated smoking intensity by primary sampling unit (PSU), leveraging its exogeneity from the higher level of aggregation, as per Deaton’s model. Since we did not have access to specific household municipalities, data were aggregated at the PSU level.

In the Table A1 in the appendix, we report the Hansen J statistics, which test the validity of the overidentifying restrictions in our IV estimation. A non-rejection of the null hypothesis indicates that the instruments are valid—that is, they are uncorrelated with the error term and correctly excluded from the estimated equation. In our case, the test results support the validity of the instruments used, reinforcing the reliability of our IV estimates.

A second challengeinvolves potential contemporaneous correlation between the error terms of the different equations in the demand system. This correlation can arise because the dependent variables in each demand equation may be affected by common shocks or omitted variables, which would violate the assumptions of independent errors across equations. While the most suitable estimation method for this issue, might be 3SLS-GMM, we encountered convergence issues. Therefore, we estimated the system using generalized 3SLS with a bootstrapped procedure (500 replications), which allows for flexibility in instrumented variables across equations. This approach controls for the contemporaneous correlation of errors while addressing the potential heteroscedasticity in the model by using a bootstrap procedure with 500 replications. We also applied GMM 2SLS with a robust covariance matrix for standard errors as an alternative method, but this did not lead to any significant change in the results.

The third challenge involves the heterogeneity in preferences between tobacco users and non-users. Non-users may have zero tobacco expenditures for different reasons: either because they cannot afford tobacco (a corner solution) or because they choose not to consume tobacco, as it does not contribute to their utility (abstention). In this latter case, the consumption preferences of tobacco users and non-users may differ across other commodity groups. To test for such heterogeneity, we examined whether the coefficients for tobacco use $\left( {\beta_{0di} , \beta_{2di} ,\beta_{3di} } \right)$ were jointly significant using the Wald test. If these coefficients are significant, it would indicate that tobacco-using and non-using households allocate their spending differently across consumption categories (see Section “Econometric model”).

### Variables

The dataset comprises 7,518 households and provides detailed statistics on budget shares allocated to various categories of consumption, as well as household characteristics and instrumental variables. Food accounts for the largest share of household budgets (50.2%), followed by housing (13.1%), and transport (5.4%). Expenditures on tobacco and alcohol are at 2.7% and 1.1% of the household budget, respectively.

Health, education, and entertainment each account for under 5% of the budget, while spending on communication and durable goods remains modest. Household average total consumption stands at 73,401 Albanian Lek (1 Euro = 98.5 ALL.), with tobacco expenditure averaging 1,729 ALL.

Households typically consist of about 3.8 members, with an average of 1.3 employed or self-employed individuals. On average, households include 0.6 members under the age of 14 and 0.5 over the age of 65, and 60% of households reside in urban areas.

The instrumental variables suggest moderate variation in household composition and smoking behavior, with an adult-to-total household member ratio of 0.7 and an average cigarette consumption intensity of 2.9 packs per PSU. Descriptive statistics of the variables can be found in Table 1.

**Table 1:** Descriptive statistics.

Description	Variable name	Mean	SE	Confidence interval lower	Confidence interval upper	N_obs
Budget share of tobacco spending	tobacco	2.7	0.07	2.6	2.9	7518
Budget share of food spending	food	50.2	0.17	49.8	50.5	7518
Budget share of health spending	health	3.7	0.08	3.5	3.8	7518
Budget share of education spending	education	1.7	0.11	1.5	1.9	7518
Budget share of housing spending	housing	13.1	0.11	12.8	13.3	7518
Budget share of clothing spending	cloths	4.0	0.06	3.8	4.1	7518
Budget share of entertainment spending	entertainment	2.4	0.06	2.3	2.6	7518
Budget share of transport spending	transport	5.4	0.10	5.2	5.6	7518
Budget share of durable goods spending	durable	5.1	0.05	5.0	5.2	7518
Budget share of communication spending	comunication	3.9	0.04	3.8	4.0	7518
Budget share of restaurant spending	restorants	3.7	0.09	3.5	3.9	7518
Budget share of alcohol spending	alcohol	1.1	0.02	1.1	1.1	7518
Instrumented variables						
Tobacco expenditures	tob_exp	1728.82	39.94063	1650.525	1807.114	7518
Non-tobacco expenditures	M	71672.56	647.0142	70404.23	72940.88	7518
Total consumption	Y	73401.37	655.0773	72117.24	74685.51	7518
Variables used to control for household characteristics						
Household size	HHsize	3.8	0.022	3.8	3.8	7518
Maximum education level in the household	maxedu	34.7	0.203	34.3	35.1	7518
Number of household members employed or self-employed	Employ_Self	1.3	0.013	1.3	1.3	7518
Number of household members under 14 years old	under14	0.6	0.014	0.6	0.6	7518
Number of household members over 65 years old	over65	0.5	0.009	0.5	0.5	7518
Dummy variable for whether household lives in urban area	urban	0.6	0.002	0.6	0.6	7518
Instrumental variables						
Number of males relative to number of females in the household	asexratio	1.2	0.011	1.2	1.3	7302
Number of adults as a share of total household size	adultratio	0.7	0.004	0.7	0.7	7518
Average number of cigarette packs consumed in PSU	intensity	2.9	0.035	2.8	3.0	7518

Data represent monthly values. Expenditures are expressed in ALL (Albanian Lek). The data are weighted using survey weights.

## Results

Table 2 provides an overview of spending patterns across different income groups (low, middle, and high) and various household expenditure categories, shedding light on how income levels shape financial priorities. Low-income households, constrained by limited resources, focus heavily on essential expenditures such as food and housing dedicating a significant share of their budget (respectively 57.5% and 17.9%), leaving minimal room for education (0.1%) or a non-essential category like entertainment (1.1%). In contrast, high-income households exhibit greater flexibility, allocating a larger share of their budgets to education (4.9) and discretionary spending, including entertainment (3.9%) and restaurants (6.4%).

**Table 2:** Average monthly budget share for all categories of expenditures by income group.

Variable	All sample	Low income	Mid income	High income
Tobacco	2.73 [2.60, 2.85]	2.13 [1.83, 2.44]	3.14 [2.94, 3.34]	2.45 [2.27, 2.64]
Food	50.16 [49.82, 50.50]	57.50 [56.89, 58.11]	52.67 [52.26, 53.09]	40.38 [39.73, 41.03]
Health	3.66 [3.50, 3.82]	3.30 [2.99, 3.61]	3.58 [3.37, 3.79]	4.08 [3.73, 4.43]
Education	1.70 [1.48, 1.91]	0.11 [0.05, 0.17]	0.54 [0.43, 0.65]	4.87 [4.16, 5.58]
Housing	13.05 [12.84, 13.27]	17.92 [17.47, 18.38]	12.97 [12.69, 13.25]	9.56 [9.18, 9.94]
Cloths	3.96 [3.84, 4.07]	2.39 [2.16, 2.62]	4.02 [3.85, 4.19]	5.01 [4.79, 5.23]
Entertainment	2.44 [2.32, 2.56]	1.06 [0.90, 1.21]	2.18 [2.03, 2.34]	3.91 [3.62, 4.21]
Transport	5.43 [5.23, 5.63]	1.74 [1.56, 1.92]	4.47 [4.22, 4.71]	9.84 [9.35, 10.33]
Durable	5.12 [5.03, 5.21]	5.15 [4.95, 5.36]	5.35 [5.22, 5.47]	4.71 [4.55, 4.87]
Other	5.78 [5.67, 5.90]	3.63 [3.41, 3.84]	6.05 [5.90, 6.20]	6.93 [6.69, 7.18]
Communication	3.90 [3.83, 3.97]	5.01 [4.82, 5.20]	3.88 [3.79, 3.98]	3.10 [3.01, 3.20]
Restaurant	3.69 [3.51, 3.87]	1.41 [1.24, 1.57]	3.12 [2.92, 3.31]	6.37 [5.87, 6.87]
Alcohol	1.10 [1.06, 1.14]	0.79 [0.70, 0.87]	1.17 [1.11, 1.23]	1.22 [1.15, 1.29]
Observations	7518	1880	3758	1880

All budget values are weighted using household survey weights. Reported means are accompanied by 95% confidence intervals in brackets.

A key observation across all income groups is the role of tobacco as an addictive expense that influences household decision-making. Despite variations in income, tobacco expenditures remain a notable share of household budgets, with mid-income households allocating the highest proportion (3.1%) compared to low- and high-income households (2.1% and 2.5%, respectively). This reflects tobacco’s persistent impact on financial choices, as it often competes with other critical spending needs. This addiction-driven spending may further reinforce economic disparities, as lower-income households, already stretched thin, are more likely to be gravely affected by the crowding-out effect, diverting resources away from other critical needs to accommodate tobacco costs.

The values identified as significant in Table 3 reveal distinct differences in spending patterns between smoking and non-smoking households, underscoring how smoking, shapes household decision-making. Non-smoking households allocate significantly more to housing and education. In contrast, smoking households spend significantly more on clothing and transport, which may reflect lifestyle choices. The most striking difference is in alcohol spending, where smoking households allocate much more, nearly double the share of their budgets, highlighting a likely association between smoking and increased alcohol consumption.

**Table 3:** Budget share of smoking and non-smoking households.

Category	Non-smoking households	Smoking households	Difference	t-stat
Food	50.4	49.8	0.5	1.427
Health	3.6	3.7	-0.1	-0.602
Education	1.9	1.4	0.5	2.069**
Housing	13.8	11.9	1.8	8.251***
Clothing	3.8	4.2	-0.3	-2.660***
Entertainment	2.3	2.6	-0.3	-2.275**
Transport	5.0	6.1	-1.1	-5.247***
Durable goods	5.0	5.3	-0.3	-3.293***
Other	5.7	5.9	-0.2	-1.920*
Communication	4.0	3.7	0.4	4.916***
Restaurants	3.6	3.8	-0.1	-0.607
Alcohol	0.8	1.5	-0.6	-13.911***

All budget values are weighted using household survey weights; * p < 0.05, ** p < 0.01, *** p < 0.001.

Table 4 presents the estimated annual crowding-out effects of tobacco expenditures on the budget shares of other spending categories for all households and by income group. These effects are expressed in percentage points and are based on a 1,200 ALL increase in annual tobacco spending. This means that an increase of 1,200 ALL per year in tobacco spending per household—equivalent to approximately 6% of the average household tobacco expenditure—would lead to the reported changes in spending shares.

**Table 4:** Estimation results for yearly crowding out effect of tobacco spending in the budget share of other expenditures categories (for all households and by income group) in percentage points (%).

Category	All households	Low income	Mid income	High income
Food	0.11***	0.25*	0.09***	0.14***
	(-8.04)	(-2.58)	(-5.29)	(-6.66)
Health	-0.02*	0.05	-0.04***	0.01
	(-2.25)	(-0.80)	(-4.33)	(-0.73)
Education	-0.04***	-0.01	-0.01*	-0.10***
	(-4.87)	(-1.46)	(-2.34)	(-4.24)
Housing	-0.02**	-0.10	-0.04***	-0.00
	(-2.80)	(-1.42)	(-3.30)	(-0.12)
Clothing	-0.02***	-0.05	-0.03***	-0.00
	(-3.65)	(-1.59)	(-4.39)	(-0.44)
Entertainment	0.01**	-0.04*	0.01*	0.01
	(-2.60)	(-1.98)	(-2.52)	(-1.44)
Transport	0.01	0.01	0.02*	-0.02
	(-1.09)	(-0.46)	(-1.96)	(-1.19)
Durable goods	0.01	0.03	0.00	0.01
	(-1.33)	(-0.83)	(-0.73)	(-0.80)
Communication	-0.00*	-0.10***	-0.00	0.00
	(-2.01)	(-3.83)	(-0.61)	(-0.83)
Restaurants	-0.04***	0.01	-0.02**	-0.06**
	(-4.57)	(-0.40)	(-3.29)	(-3.22)
Alcohol	0.02***	-0.02	0.02***	0.02***
	(-5.96)	(-1.46)	(-5.64)	(-5.13)
N	7302	1761	3684	1857

t statistics in parentheses, * p < 0.05, ** p < 0.01, *** p < 0.001; Each coefficient reflects the yearly percentage point change in a category’s share of the total budget per 1 ALL increase in tobacco expenditure.

For all households, a 1,200 ALL increase in tobacco spending is associated with a 0.11 percentage point increase in the share of the household budget allocated to food. For a household that initially spends 30% of its budget on food, this would increase to approximately 30.11%. At the same time, the shares allocated to health and education decline by approximately 0.02 and 0.04 percentage points, respectively. For example, a household allocating 5% of its budget to education would see that share fall to about 4.96%. Similar reductions are observed in spending on housing, clothing, communication, and restaurants. These patterns reflect a reallocation of household resources away from long-term welfare-oriented categories toward more immediate consumption, suggesting that tobacco use not only imposes direct health costs but also indirectly erodes household investment in human capital and well-being.

When disaggregated by income group, the overall pattern of crowding out is primarily driven by middle-income households. In this group, increased tobacco spending significantly reduces budget shares allocated to health, housing, education, and clothing, while increasing spending on food, alcohol, and entertainment. These changes mirror the aggregate findings and suggest that middle-income households bear much of the reallocation burden associated with smoking.

In low-income households, tobacco expenditure increases are associated with a decrease in spending on communication and entertainment, while the share of income allocated to food rises. However, caution is warranted in interpreting these results, as the model’s fit for this group is weaker, and estimates may be less reliable.

Among high-income households, the results show reductions in budget shares for education and restaurant spending following an increase in tobacco expenditure. This may suggest a reallocation away from non-essential or discretionary services. Simultaneously, food and alcohol spending increase, consistent with patterns observed in other income groups. These shifts indicate that across the income spectrum, smoking tends to redirect household resources toward immediate consumption, particularly food and alcohol, often at the expense of education, health, and other socially beneficial expenditures.

To better understand the heterogeneity in the crowding-out effects of tobacco spending, we extended the analysis to include geographic regions and the rural–urban divide. The results are presented in Table A2 in the appendix. The effects vary substantially across Albanian regions. In the Center, the impact is most pronounced: tobacco spending reduces the budget share allocated to health by 0.06 percentage points, to education by 0.04, and to housing by 0.06, while increasing food spending by 0.18 and entertainment by 0.03. The South shows a similar pattern, though somewhat less intense, with food increasing by 0.07 and education declining by 0.05. In contrast, the North exhibits weaker and largely insignificant effects, with only modest shifts such as a 0.04 increase in food and a 0.03 reduction in restaurant spending.

Differences between urban and rural areas are also evident. Urban households show stronger crowding-out effects: spending on health falls by 0.03, education by 0.04, and restaurants by 0.06, while food spending rises by 0.17. In rural households, the effects are milder, with a 0.06 increase in food, a 0.02 decrease in clothing, and a 0.03 rise in alcohol spending. These patterns suggest that urban and Center-region households are more likely to reallocate resources away from welfare-enhancing categories toward basic consumption when tobacco spending increases.

To test for heterogeneity in spending behavior based on smoking status, we estimated Eq. (2) and examined whether the coefficients associated with tobacco use— specifically the interaction terms $\beta_{0di} , \beta_{2di} ,\beta_{3di}$ — were jointly significant across key spending categories. We used a Wald test to assess the joint significance of these coefficients. The results of the Wald test indicated statistically significant heterogeneity at 5% level for health, housing, and clothing, suggesting that smoking status influences how households allocate their budgets in these categories. In other words, smokers and non-smokers appear to exhibit systematically different spending patterns for these goods, conditional on tobacco use. However, for the remaining categories, the Wald tests did not reject the null hypothesis of joint insignificance, indicating that the tobacco-related interaction terms did not significantly improve the model’s explanatory power. In other words, when the Wald test was applied to these categories (e.g., food, transport, entertainment), it did not provide sufficient evidence to conclude that smokers and non-smokers allocate their budgets differently in a statistically meaningful way, once income and other household characteristics are taken into account. This suggests that while smoking may affect the overall household budget, it does not necessarily reflect a change in preferences for these specific goods. Because the coefficients for tobacco expenditures were not jointly significant across the full set of categories, and to maintain clarity and focus in the presentation of results, we do not report these estimates in the main tables.

## Discussion and conclusions

Tobacco consumption has a direct and profound impact on household decision-making by creating immediate opportunity costs. Funds spent on tobacco are diverted from other essential goods and services, limiting households’ ability to invest in critical needs such as healthcare, education, and basic necessities. Indeed, our research findings show that tobacco spending decreases expenditure on essentials including health, education, housing and clothing.

Beyond its monetary implications, tobacco use is often linked to changes in other health-related behaviors, such as alcohol consumption and dietary habits^45^. Indeed, our study identifies a positive relationship between tobacco and alcohol expenditures in Albania, consistent with prior research^16–18,45^. Similarly, we found a positive association between tobacco and food expenditures similar to^10^. Unfortunately, due to the lack of disaggregated data on food spending, it remains unclear whether this reflects a preference for processed or less healthy foods. Previous studies have highlighted a tendency for tobacco spending to correlate with higher processed food consumption but lower expenditure on healthier foods consumed at home. Improved disaggregation of food expenditure data in future surveys could help clarify this relationship.

Another limitation of the study, is that it does not distinguish between tobacco and other forms such as e-cigarettes. When the relevant consumption data become available, future studies should address given the increasing consumption of e-cigarettes and other tobacco products as documented by recent studies^23^. Furthermore, because the data are collected at the household level, it is not possible to identify which specific categories of household members—such as children, the elderly, or women—are most affected by the crowding-out effect of tobacco expenditure. The aggregated nature of the data masks intra-household allocation patterns, limiting our ability to assess the differential impact on individual household members.

Another limitation is linked to the limited timespan – our study relies on one year cross-sectional data (survey), different from some other studies which had the luxury of more years of data covering longer time spans^44^. Consequently the findings should not be interpreted as causal. Although the use of instrumental variables helps mitigate endogeneity concerns, the data are observational and cross-sectional, which limits causal inference. The relationships identified between tobacco expenditure and other household spending patterns should be interpreted as associations that are robust to a range of controls. Future research using multi-year or longitudinal data would allow for stronger causal analysis. Lastly, the findings rely on self-reported household data, which may be subject to underreporting—particularly for behaviors like smoking, which are considered addictive and potentially sensitive. Although underreporting of tobacco use has historically been relatively limited, recent evidence suggests it may be increasing over time^46^.

The findings underscore the urgent need for targeted tobacco control measures to mitigate the crowding-out effects observed in household expenditures. Increasing excise taxes on tobacco products would raise the cost of smoking, potentially deterring consumption and freeing up resources for essential categories such as health, education, and housing. Research demonstrates that higher tobacco excise taxes, which increase cigarette prices, reduce smoking initiation rates among teenagers^14^ and encourage cessation among adults. This approach not only curtails tobacco use but also indirectly reduces teen drinking^16^, creating dual benefits from such fiscal policies. Additionally, public awareness campaigns and behavioral support programs can address the addictive nature of tobacco use, helping households make more balanced financial decisions. Policymakers should also focus on supporting low- and middle-income households, where the financial strain of tobacco consumption is most severe, to ensure resources are directed toward improving long-term well-being and economic stability.

## Electronic supplementary material

Below is the link to the electronic supplementary material.

Supplementary Material 1

## Data Availability

The data that support the findings of this study are available from INSTAT Albania but restrictions apply to the availability of these data, which were used under license for the current study. Data are however available from INSTAT Albania upon request.
