# Supplementary material for: The dual impact of tobacco spending: crowding out essentials and crowding in addictive behaviors
Source: Sci Rep. 2025 Jul 9;15:24648. doi: 10.1038/s41598-025-08648-1 (PMC12241404; doi:10.1038/s41598-025-08648-1)
Supplement: Supplementary file 1 — Supplementary Material 1 [file 41598_2025_8648_MOESM1_ESM.docx]

# Appendix

**Table A1.** Tests of hetroskedasticity and validity of instruments

| **Category** | **Pagan Hall** | **p_values** | **Hansen J** | **p-values** | **Instruments** |
| --- | --- | --- | --- | --- | --- |
| food | 148.972 | 0.000 | 2.303 | 0.129 | sexratio, intesity |
| health | 359.466 | 0.000 | 2.152 | 0.142 | sexratio, intesity |
| education | 1478.696 | 0.000 | 0.639 | 0.424 | sexratio, adultratio |
| housing | 107.008 | 0.000 | 3.919 | 0.048 | sexratio, intesity |
| cloths | 141.962 | 0.000 | 0.363 | 0.547 | sexratio, intesity |
| entertainment | 104.168 | 0.000 | 1.372 | 0.241 | sexratio, intesity |
| transport | 383.821 | 0.000 | 1.502 | 0.220 | sexratio, adultratio |
| durable | 63.249 | 0.000 | 2.084 | 0.149 | sexratio, intesity |
| communication | 225.241 | 0.000 | 2.288 | 0.130 | sexratio, intesity |
| restaurants | 612.038 | 0.000 | 5.364 | 0.021 | sexratio, intesity |
| alcohol | 117.912 | 0.000 | 0.700 | 0.403 | sexratio, adultratio |

**Table A2:** Crowding-Out Effects of Tobacco Expenditure in other goods budget share by Region (North, Center, South) and Area Type (Rural vs. Urban) in percentage points of the total budget (%)

|  | All households | North | Center | South | Rural | Urban |
| --- | --- | --- | --- | --- | --- | --- |
| food | 0.1116*** | 0.0420* | 0.1800*** | 0.0732** | 0.0636*** | 0.1668*** |
|  | (8.865) | (1.989) | (8.243) | (2.579) | (3.509) | (9.804) |
| health | -0.0180* | 0.0048 | -0.0576*** | 0.0444* | -0.0060 | -0.0348** |
|  | (-2.157) | (0.480) | (-4.730) | (2.377) | (-0.503) | (-3.105) |
| education | -0.0444*** | -0.0228 | -0.0396*** | -0.0492* | -0.0504*** | -0.0384** |
|  | (-5.705) | (-1.950) | (-3.641) | (-2.013) | (-3.986) | (-2.979) |
| housing | -0.0252** | -0.0192 | -0.0540*** | -0.0468* | -0.0276 | -0.0216 |
|  | (-3.191) | (-1.457) | (-4.001) | (-2.504) | (-1.851) | (-1.654) |
| cloths | -0.0168*** | -0.0084 | -0.0288*** | 0.0156 | -0.0204** | -0.0132* |
|  | (-3.340) | (-1.262) | (-3.908) | (1.570) | (-2.847) | (-1.996) |
| entertainment | 0.0120** | 0.0036 | 0.0312*** | -0.0060 | 0.0192** | 0.0060 |
|  | (2.631) | (0.725) | (4.747) | (-0.631) | (2.608) | (1.309) |
| transport | 0.0096 | 0.0456*** | -0.0348** | -0.0132 | 0.0288 | -0.0108 |
|  | (1.165) | (3.727) | (-2.774) | (-0.641) | (1.891) | (-0.912) |
| durable | 0.0060 | -0.0096 | 0.0120 | 0.0252* | -0.0084 | 0.0168** |
|  | (1.342) | (-1.521) | (1.690) | (2.294) | (-1.478) | (2.816) |
| communication | -0.0048 | -0.0132** | 0.0036 | -0.0192** | -0.0072 | -0.0024 |
|  | (-1.754) | (-2.895) | (0.875) | (-2.973) | (-1.757) | (-0.566) |
| restaurants | -0.0384*** | -0.0300*** | -0.0372*** | -0.0276 | -0.0264* | -0.0564*** |
|  | (-5.013) | (-3.401) | (-3.919) | (-1.339) | (-2.543) | (-4.828) |
| alcohol | 0.0156*** | 0.0012 | 0.0348*** | 0.0144*** | 0.0300*** | 0.0024 |
|  | (6.611) | (0.445) | (8.615) | (3.298) | (8.256) | (0.917) |
| R2 | 0.298896 | 0.240454 | 0.27944 | 0.298287 | 0.317091 | 0.269876 |
| N | 7302 | 2022 | 2506 | 2774 | 3544 | 3758 |

**Note:** t-statistics are reported in parentheses. Standard errors were bootstrapped using 100 replications.
